# Investigating Patient Satisfaction Through Web-Based Reviews of Norwegian Dentists: Quantitative Study Using the Meaning Extraction Method

**DOI:** 10.2196/49262

**Published:** 2024-05-03

**Authors:** Maria Larsen, Gro Eirin Holde, Jan-Are Kolset Johnsen

**Affiliations:** 1 Department of Clinical Dentistry Faculty of Health Sciences UiT The Arctic University of Norway Tromsø Norway; 2 The Public Dental Health Service Competence Centre of Northern Norway Tromsø Norway

**Keywords:** internet use, Linguistic Inquiry and Word Count, LIWC, patient satisfaction, patient preference, challenging encounters, preventive dentistry, population surveillance

## Abstract

**Background:**

Challenging encounters in health care professions, including in dentistry, are relatively common. Challenging encounters can be defined as stressful or emotional situations involving patients that could impact both treatment outcomes and patients’ experiences. Through written web-based reviews, patients can share their experiences with health care providers, and these posts can be a useful source for investigating patient satisfaction and their experiences of challenging encounters.

**Objective:**

This study aims to identify dominant themes from patient-written, web-based reviews of dentists and investigate how these themes are related to patient satisfaction with dental treatment.

**Methods:**

The study data consisted of 11,764 reviews written by dental patients, which included 1- to 5-star ratings on overall satisfaction and free-text comments. The free-text comments were analyzed using Linguistic Inquiry and Word Count software, and the meaning extraction method was used to group words into thematic categories. These themes were used as variables in a multilevel logistic regression analysis to predict patient satisfaction.

**Results:**

Eight themes emerged from the analyses, of which 6 (75%)—*explanation* (odds ratio [OR] 2.56, 95% CI 2.16-3.04; *P*<.001), *assurance* (OR 3.61, 95% CI 2.57-5.06; *P*<.001), *performance assessment* (OR 2.17, 95% CI 1.84-2.55; *P*<.001), *professional advice* (OR 1.81, 95% CI 1.55-2.13; *P*<.001), *facilities* (OR 1.78, 95% CI 1.08-2.91; *P*=.02), and *recommendation* (OR 1.31, 95% CI 1.12-1.53; *P*<.001)—increased the odds of high patient satisfaction. The remaining themes (2/8, 25%)—*consequences of treatment need* (OR 0.24, 95% CI 0.20-0.29; *P*<.001) and *patient-centered care* (OR 0.62, 95% CI 0.52-0.74; *P*<.001)—reduced the odds of high patient satisfaction.

**Conclusions:**

The meaning extraction method is an interesting approach to explore patients’ written accounts of encounters with dental health professionals. The experiences described by patients provide insight into key elements related to patient satisfaction that can be used in the education of dental health professionals and to improve the provision of dental health services.

## Introduction

### Challenging Encounters and Patient Satisfaction

Challenging encounters in health care are not uncommon [1,2] and can be defined in various ways by individual health care providers [3]. Situations such as dealing with violent patients, “breaking bad news,” and managing demanding family members are examples of challenging situations mentioned by health care providers [4,5]. Health care providers have also referred to patients they perceive as challenging as the source of conflict, and anxious or angry patients are most often mentioned as challenging [4,6]. Studies have also defined challenging encounters as situations where patients are “causing negative feelings in physicians” [7], and challenging encounters between health care providers and patients have been linked to increased burnout and stress among health care providers [2,8]. This issue is also highly relevant in dentistry, and dentists have reported that up to 25% of their daily encounters are perceived as challenging [6]. While studies have addressed how health care providers define and experience challenging encounters, the patient experience has been explored to a lesser extent. Whether a patient has experienced a challenging encounter could be researched through use of patient satisfaction measures.

Patient satisfaction has been defined in many different ways in research through the years. In a recent literature review [9], three main definitions of patient satisfaction were identified: (1) the patients’ experience based on their expectations of a health care service and how the service met their expectations, (2) patient satisfaction defined as feedback forming the basis for the improvement of health care services, and (3) patient satisfaction defined as the patients’ perception of health care providers’ ability to provide proper care and the quality of the interpersonal relationship. Research investigating patient satisfaction has revealed inconsistent results regarding the establishment of important determinants [10]. This might be due to differing definitions of the concept of patient satisfaction among studies [9,10]. In addition, in most studies, patient satisfaction is generally reported as high; however, this could be an overstatement due to limitations in the data collection instruments, and it has been suggested that including measurements of patient dissatisfaction in the instruments may help produce a more correct picture [10]. Arguably, there is a need to include issues relevant to patients that are not predefined by health care personnel or researchers [9]. A recent literature review has criticized current methods for evaluating patient satisfaction in health care, arguing that they seem to have been adopted from consumer satisfaction models and could therefore be inappropriate in health care settings [9]. Research has shown that the most important predictor of patient satisfaction is high-quality patient communication [11]. In addition, what seems most important to patients when indicating satisfaction is the relationship between the patient and the health care personnel, as well as the perceived social abilities of the health care personnel [12]. Furthermore, a link has been found between patient satisfaction and the performance of dental health professionals [13]. Other issues, such as the availability of services (including short waiting times, access to local hospitals, and ample parking) and the technical performance of the health care personnel, seem to matter less while still remaining important determinants of patient satisfaction [12].

### Internet Research and Health Care Services

The internet provides almost unlimited user-generated content available for research, and for health researchers, it presents the opportunity to investigate the general public’s opinions and knowledge on a myriad of topics, including those related to health [14,15]. These data also enable research on social interactions (eg, the interactions between caregivers and users in web-based treatment procedures [15] through the use of natural language processing [NLP] [16]). NLP refers to the use of computational models on natural text materials to study associations between language and other variables, including the prediction of behavior or other outcomes. It is used widely in several disciplines (eg, opinion mining in sales and marketing services [14] as well as research on user-written reviews of experiences and products [17]). The methods within the field of NLP can also be used to investigate interesting health-related aspects, such as the detection of signs of clinical depression [18] and social anxiety [19]. In the broader context of health care, topics such as users’ opinions, experiences, and health literacy and competence are relevant to investigate with NLP [15]. In addition, patient-written reviews of health care services could present a major source of information relevant to health care workers.

There are currently many websites that provide patients with the opportunity to rate and write about their experiences with health care providers. While the use of social media as a platform for health communication is generally considered a powerful tool for both patient and health care providers [20], web-based reviews of health care services and health care providers might provide unique insight into the experiences of patients and their evaluations of the quality of health services [20]; for example, in recent research investigating the web-based reviews of an obstetric care clinic, it was found that patients’ experiences of the quality of the facilities and the perception of staff as comforting and providing high-quality care were associated with increased patient satisfaction [21]. Compared to other means of providing feedback to health care professionals, web-based reviews have benefits such as perceived anonymity and freedom from potentially negative consequences of evaluating figures of authority. Further benefits can be related to the social dimension of disseminating one’s views, experiences, and opinions to peers [20]. However, some challenges are also apparent, such as the subjectivity and contextual nature of web-based reviews [22] as has been found for other web-based evaluations related to health care [23].

### Web-Based Evaluations of Dentists

While numerous studies have examined web-based ratings of physicians [9], few have examined web-based ratings of dentists. In a study of web-based evaluations of dentists in Germany, it was found that rating scores were largely positive and that younger or female dentists provided the most positive ratings [24]. In addition, differences in ratings emerged among clinical specialties, with pediatric dentists receiving better ratings than orthodontists [24]. Furthermore, a study published in the United States showed that younger or female dentists received the best web-based reviews, as did dentists where patients experienced shorter waiting times [25]. Studies also point to specific topics that seem to influence evaluations, such as experiences of discomfort perceptions of a lack of professional ethics [25], and topics that might be specifically related to challenging encounters or negative evaluations of dentists on the web [26]. Interestingly, negative web-based reviews about dentists, while uncommon [24,26], are perceived as more trustworthy than positive reviews [27].

Considering these findings and challenges, this study aims to investigate how the content of web-based reviews of dentists in Norway can be used to predict patient satisfaction and challenging encounters, indicated by high and low rating scores, respectively, through the following steps: (1) identify dominant themes discussed by patients in their reviews and (2) investigate the dominant themes and their relationship with patient satisfaction and challenging encounters as rated by the patients.

## Methods

### Overview

The data were extracted from a Norwegian website that helps patients connect with health care professionals by the administrators of the website and made available to us as a downloadable data dump [28]. On this website, there is an option for patients to write about their experiences regarding receiving health care from dentists, general practitioners, physical therapists, and other health care professionals. A total of 11,764 patient reviews of dentists posted during the period from February 2013 to June 2020 were included in the data set. The patients rated their overall experience using 1 to 5 stars (1=*very unsatisfied* and 5=*very satisfied*) in addition to providing written comments. Patients could also rate other aspects of treatment, such as service, price, and treatment comfort. In addition, information about the date of the post and self-reported visiting frequencies was included. However, in this study, only the written comments and overall rating scores were used in the analysis.

### Language Analysis and Theme Extraction

The language analysis tool Linguistic Inquiry and Word Count (LIWC; version 2022) [29] was used to analyze the text data. The LIWC is designed to measure psychometric properties in language. As noted by Boyd [30], LIWC analysis typically works best with texts exceeding 50 words (shorter texts with a minimum of 10 words may still yield some insights, but the results may be less accurate). This is because LIWC dictionaries work by calculating the relative percentage of a word’s occurrence in a body of text. In our analysis, the Norwegian LIWC 2007 dictionary was used [31]. By applying the meaning extraction method (MEM) through the LIWC's built-in meaning extraction helper, we could determine the dominant word categories used in the reviews. A detailed description of the principles behind the MEM can be found elsewhere [30]; however, in the following subsections, we will describe the process in detail as it relates to this data set.

### Analysis Inclusion Criteria: Text Length and Word Frequencies

The free text of the comments section of the 11,764 reviews was run through the meaning extraction helper. Each review consisted of a header and a main comment. In the analysis, all words with raw frequency of >2% were retained. The decision to use 2% instead of 5%, as recommended by Boyd [30], was due to the large number of small texts in our data set. Specifically, we found that a large number of words would appear in <5% of the material because each comment was analyzed as a single text. Hence, a 5% cutoff would exclude too many words, whereas the cutoff value of 2% provided sufficient removal of uncommon words. Each comment posted on the aforementioned Norwegian website needed to be at least 100 characters long, including punctuations and spaces. Even so, to avoid including text that would not provide any meaningful information to the content analysis (eg, exclamatory remarks such as “Great dentist!” with no further information other than signs or emojis), the inclusion criterion for the length of reviews included in the analysis was set to >5 words. To ensure meaningful results, the header was removed from further analysis because it often duplicated words used in the main comment. This could have created a false emphasis on certain commonly used phrases.

### Lemmatization List and Stop List

The MEM relies on the process of lemmatization, which requires a lemmatization list and a stop list. These were created following the recommendations from previous research [30,32,33]. The lemmatization list converts commonly used words to their word stem to count words correctly (Multimedia Appendix 1). The stop list omits words from further analysis, and the words chosen to be omitted would typically be words that were of no interest to the research question, such as the names of geographic locations, the word “dentist” (as we would expect it to be present in almost all comments), or numerical words. In addition, some function words, such as selected personal pronouns, conjunctions, and prepositions, were omitted ahead of analysis because they appeared often and could therefore dilute important content words. Examples of function words and other words omitted can be found in the stop list (Multimedia Appendix 2). Words included in the analysis were verbs, adjectives, adverbs, nouns, and all function words that were not included in the stop list. Care was taken not to omit too many words to preserve the rawness of the data. In addition, because internet-based language often adopts an informal, conversational style, resembling speech [34], we needed some function words to be retained, although some recommend that they be removed completely [30].

### Exploratory Factor Analysis

The results provided from the MEM were used to perform an exploratory factor analysis [35] using SPSS (version 28.0; IBM Corp). The MEM analysis provided a binary matrix for all reviews, which included a value of 1 if the words appeared in the review and 0 if not. The Bartlett test of sphericity and the Kaiser-Meyer-Olkin test of sampling adequacy were performed to test whether the MEM results were suitable for factor analysis. Varimax rotation was used to extract uncorrelated factor items with a factor loading threshold set to >0.2 based on the recommendations made by Markowitz [33]. Determining the number of factors to extract was based partly on an inspection of the scree plot (ie, the identification of the elbow of the plot) and eigenvalues (>1), as well as on the proposed factors’ interpretability. Words that had cross-loadings of >0.2 were omitted.

The words contained within the factors were then added to the Norwegian LIWC dictionary [31] as separate word categories. The complete data set was run through the LIWC analysis using the modified dictionary. The LIWC gives information for each review in terms of the percentage of words that matches the dictionary word categories.

### Multilevel Logistic Regression Analysis

To determine how the retrieved factors could predict patient satisfaction, a 2-level (dentist and review) random intercept logistic regression model was built, with *high patient satisfaction* as the outcome. *Patient satisfaction* was the overall rating variable recoded to a binary variable, whereby ratings of either 4 or 5 stars signified *high patient satisfaction*, and ratings of 1, 2, or 3 stars signified *low patient satisfaction*. The 8 factors (the aforementioned 8 themes) were entered as covariates recoded into binary variables—*frequent use* versus *infrequent use* or *use* versus *no use*—with the median as cutoff value (with median=0 being recoded as *no use*). A multilevel analysis was chosen as the reviews were not statistically independent variables because they could be commenting on the same dentist. The multilevel logistic regression analysis was performed in MLwiN (Centre for Multilevel Modelling, University of Bristol) [36]. The results are reported as regression coefficients, odds ratios (ORs), and respective 95% CIs. The variance partition coefficient (VPC) was also reported. The VPC estimates the proportion of the total variance in positive versus challenging encounters attributable to differences among dentists. The VPC is given as σ^2^_υ0_/(σ^2^_υ0_+Π^2^/3) [37].

### Ethical Considerations

All reviews were posted on the Norwegian website [28] voluntarily, and the data set provided by the website administrators contained only anonymous data. The study was approved by the Norwegian Centre for Research Data (468642).

## Results

### Overview of the Data

A description of the demographics of the data set can be viewed in Table 1. The mean word count of each review was 48.9 (SD 39). More than nine-tenths of the reviews (10,977/11,764, 93.31%) had a high rating score (4-5 stars), whereas the remaining reviews (687/11,764, 5.84%) had a low rating score (1-2 stars). A total of 2950 dentists had received a rating in our data set, and the mean number of reviews per dentist was 3.9.

**Table 1:** Age and sex distribution of dentists and patients.

			Dentists (n=2950), n (%)		Patients (n=11,764), n (%)
**Age (y)**					
	<20	0 (0)		64 (0.54)	
	20-30	74 (2.51)		2017 (17.15)	
	31-40	710 (24.07)		1947 (16.55)	
	41-50	823 (27.90)		1417 (12.05)	
	51-60	591 (20.03)		1098 (9.33)	
	>60	751 (25.46)		755 (6.42)	
	Missing	1 (0.03)		4466 (37.96)	
**Sex**					
	Male	1597 (54.14)		3407 (28.96)	
	Female	1328 (45.02)		4235 (36)	
	Missing	25 (0.85)		4122 (35.04)	

### Exploratory Factor Analysis

The exploratory factor analysis identified 8 factors (Textbox 1) that will be described in the following subsection. The Bartlett test of sphericity was significant (*P*<.001), and the Kaiser-Meyer-Olkin measure of sampling adequacy was 0.66. The factors extracted together explained 13.2% of the sample variation, and they were thematically labeled based on a theoretical understanding of the words they contained: *consequences of treatment need*, *explanation*, *assurance*, *facilities*, *recommendation*, *patient-centered care*, *professional advice*, and *performance assessment*.

**Factors and factor loading (%) for words from the exploratory factor analysis.**

Consequences of treatment need (eigenvalue: 3.205)

- Receive: 0.479
- Tooth: 0.478
- Must: 0.421
- Become: 0.421
- Come: 0.356
- Go: 0.350
- Caries: 0.314
- Sat: 0.296
- Because of: 0.283
- Back: 0.274
- Bad: 0.273
- Same: 0.261
- Ache: 0.258
- New: 0.254
- Wanted: 0.249
- Day: 0.244
- Pain: 0.242
- Anesthetics: 0.241
- Where: 0.239
- Enough: 0.225

Explanation (eigenvalue: 2.087)

- To do: 0.632
- Explain: 0.571
- Why: 0.386
- Good: 0.258
- Tell: 0.249
- Thorough: 0.205

Assurance (eigenvalue: 1.863)

- Feel: 0.881
- Safe: 0.676
- Take care of: 0.613
- Hands: 0.369

Recommendation (eigenvalue: 1.777)

- Recommend: 0.787
- Strongly: 0.484
- Warm: 0.466
- Could: 0.375
- Absolutely: 0.230
- Really: 0.226
- Unbelievable: 0.201

Facilities (eigenvalue: 1.671)

- Modern: 0.774
- Equipment: 0.751
- Premises: 0.507

Patient-centered care (eigenvalue: 1.571)

- Take: 0.789
- Consideration: 0.453
- Care: 0.433
- Patient: 0.263

Professional advice (eigenvalue: 1.511)

- Give: 0.505
- Advice: 0.403
- Information: 0.337
- Treatment: 0.325
- Very: 0.263
- Profoundly: 0.223
- Pleased: 0.217
- Amazing: −0.230
- Professional: 0.200

Performance assessment (eigenvalue: 1.456)

- Quick: 0.495
- Efficient: 0.428
- Nice: 0.348
- Wisdom tooth: 0.260
- Job: 0.241
- Forthcoming: 0.234

### Dominant Themes Identified by the Analysis

#### Consequences of Treatment Need

The theme *consequences of treatment need* seemed to contain words related to the patients’ need for treatment, with mentions of dental health issues such as dental caries (“tooth” and “caries”). In addition, other words associated with this theme seemed to express the urgent need to obtain an appointment (“must,” “receive,” “new,” “come,” and “go”), as well as words that might be related to an explanation of what happened (“back,” “because of,” “same,” and “where”). The word “must” could be related to the feeling of a lack of self-agency and self-determination in the situation, for example, in this quote, where the patient might have felt that they had no control of the situation:

When I first got there, she seemed friendly, but that was before the treatment started. During treatment she had no consideration and continued even though I was crying in the chair.

Example 1

In this theme, many words were action related (verbs), in the sense that something happened or certain actions were performed (“go,” “receive,” and “become”); for instance, patients would sometimes explain the turn of events resulting in a dentist appointment or their reasons for either seeking dental treatment or writing about the dental encounter. Arguably, it could also be the case that these words were related to the feeling of unmet expectations (“wanted” and “enough”). Typically, patients would often describe themselves as experiencing dental anxiety, which contributed to an uncomfortable treatment situation:

He got annoyed and asked very rudely what my problem was. Well yeah mister I have dental phobia! DO YOU EVEN KNOW SOME PEOPLE SUFFER FROM THIS? I stopped the treatment and paid 450 NOK for him to be rude to me. Still on the lookout for a good dentist who can deal with people like me. Don’t go to him if you have this phobia!

Example 2

#### Explanation

The theme *explanation* contained words such as “explain,” “tell,” and “why.” From the other prevalent words in this theme (“thorough,” “good,” and “to do”), it could be argued that patients used these words to describe instances where the dentist thoroughly explained the treatment or other topics, as exemplified by this quote:

[Name] adapts the treatment, stops and gives you small breaks during treatment, check that you feel okay, she is very good at explaining what is going to happen and what she does during treatment.

Example 3

#### Assurance

The theme *assurance* contained words related to safety and care (“safe,” “take care of,” “hands,” and “feel”) as experienced in relation to the encounter between patient and dentist:

You feel like you are in good hands. A cheerful and pleasant lady! Your dental fear disappears when you sit down and she begins to talk.

Example 4

#### Facilities

The theme *facilities* contained the words “modern,” “equipment,” and “premises,” which indicates that patients specifically noticed the environment of the dental clinic:

Shows and explains to you using modern equipment. I strongly recommend him.

Example 5

Got no information about cost and got yelled at for not using them last time (dental emergency office—I have a regular dentist) bragged about the expensive equipment, where I had to pay 900 NOK for a picture I didn’t need.

Example 6

#### Recommendation

The theme *recommendation* contained words related to the need to disseminate the patients’ views of the dentist to others, with words such as “recommend” and “strongly”:

I recommend him to everyone I know with toothache.

Example 7

I strongly recommend this dentist!

Example 8

#### Patient-Centered Care

The theme *patient-centered care* contained words related to patient-centered care or the experience of empathetic behavior from the dentist (“take,” “care,” “patient,” and “consideration”). It would be tempting to think that this theme would be linked to *high patient satisfaction*, but the words could also be used to express how the patient would have liked to be treated; for instance, in the following quote, we see how the words related to patient-centered care were used when the patient expressed experiencing a lack of patient centeredness:

It is distressing that there are dentists that have so little consideration for their patients. When you are in a vulnerable situation beforehand, then this is the last thing you need. It is not just teeth they are working with, but humans!

Example 9

In any case, we noted that patients often wrote about patient centeredness and found it important to experience that the staff and dentist were comforting.

#### Performance Assessment

The theme *performance assessment* contained words describing the perceived performance of the dentist, an inference to how they performed and the quality of the performance. Here, we find words such as “nice,” “quick,” “efficient,” and “wisdom tooth.” Patients writing the reviews seemed to value their time, and efficient dentists (those completing procedures quickly) were viewed more favorably than dentists perceived to be inefficient at managing their time:

Removed all 4 of my wisdom teeth in a total of 31 (!!!) minutes. 18 minutes the first time and 13 minutes the second time. Do I have to say more? Great experience!

Example 10

I was not impressed when I went to [name]. I think he spends too much time treating relatively simple issues. Had some complications with a dental restoration that he did which never really got better.

Example 11

Interestingly, dentists who were perceived as careless or too quick may risk increasing the likelihood of posttreatment issues for patients:

Rushed through the appointment, did not wait long enough to let the anesthetics kick in and drilled right into the nerve, so my head exploded. My dental anxiety that [name; in the same building] had cured came back.

Example 12

Patients value high-quality work and might feel more pleased with treatment if the dentist acts professionally, is competent, and achieves efficiency without compromising the quality of the treatment.

#### Professional Advice

The theme *professional advice* consisted of words related to providing information and clinical advice to patients, such as “give,” “advice,” and “information.” It also contained quality assessments of how the advice was perceived or provided, as we can infer from the words “amazing,” “professional,” “pleased,” “very,” and “profoundly.” Patients clearly appreciate professional advice on how to take care of their oral health and their treatment options:

Experience this dentist as skilled, thorough and detail oriented. Gives good information about follow up treatment and what to do at home.

Example 13

[Name]’s ability to inform about how to treat the post-treatment complications was bad, and the recommended measures had no effect.

Example 14

Professional, nice and efficient. Good at explaining and I felt safe and taken care of. I got sufficient information ahead of treatment on recommended procedures. Was happy with their follow up on me during treatment and afterwards as well, and how efficient and professional the work was done.

Example 15

In addition, patients would sometimes express concerns about professionalism, for instance, when they perceived that the personal beliefs of dentists were indistinguishable from professional medical advice:

She tried to push life-threatening antivaccination propaganda on me, without me even bringing up the subject, and what in God’s grace does a dentist know about vaccines? And be careful with the double standards all the time she offers Botox treatment (Botox is a nerve toxin).

Example 16

### Predicting Patient Satisfaction

**Figure 1:**
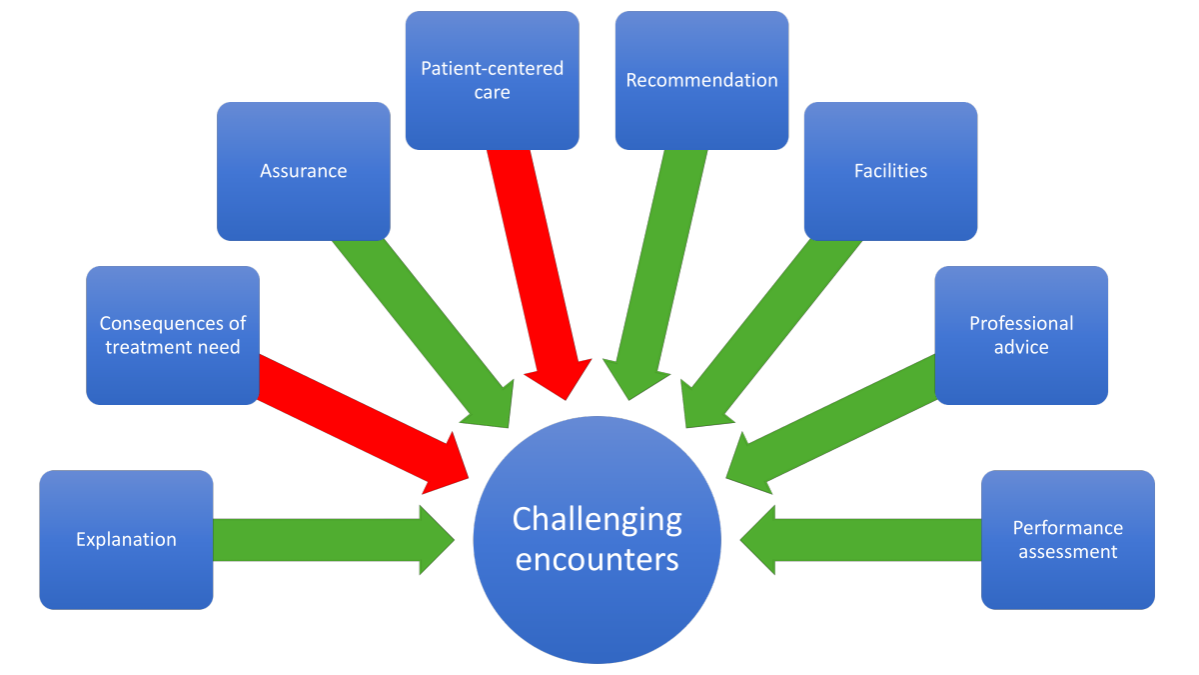
The dominant themes identified and their relation to the challenging encounter. The green arrows indicate that the factor decreases the likelihood of a challenging encounter, while the red arrows indicate that the factor increases the likelihood of a challenging encounter.

To predict patient satisfaction based on the dominant themes, a multilevel logistic regression analysis was performed (Table 2; Figure 1). Of the total explained variance, 28% was attributable to the differences among dentists (VPC=0.28).

The regression analysis showed that when the patients used words related to *explanation*, the OR for a high satisfaction score (4 or 5 stars) was 2.56 (95% CI 2.16-3.04; *P*<.001). In addition, if words related to *assurance* were used, the OR was even higher (3.61, 95% CI 2.57-5.06; *P*<.001) for a high satisfaction rating. The odds of a high satisfaction rating also increased with the frequent use of words related to *facilities*, *professional advice*, and *performance assessment* by a factor of 1.77 (95% CI 1.08-2.91; *P*=.02), 1.81 (95% CI 1.55-2.13; *P*<.001) and 2.16 (95% CI 1.84-2.55; *P*<.001), respectively, compared to infrequent use of the respective word categories. This was also the case if patients used words connected to the theme *recommendation*, which increased the odds of the patient being satisfied with dental treatment by 31% compared to when no words related to *recommendation* were used (*P*<.001). By contrast, when patients used words related to the *patient-centered care* theme, the odds of a high satisfaction rating were reduced by 38% (*P*<.001). Similarly, for the theme *consequences of treatment need*, the frequent use of words connected to this theme reduced the odds of a high satisfaction rating by 76% (*P*<.001).

**Table 2:** A multilevel logistic regression analysis predicting patient satisfaction from dominant themes.

			B (SE)		Odds ratio^a^ (95% CI)		*P* value
**Fixed effects**							
	Intercept, *β*_0j_	2.47 (0.11)		N/A^b^		N/A	
**Themes**							
	Consequences of treatment need (frequently used vs infrequently used)	−1.43 (0.09)		0.24 (0.20-0.29)		<.001	
	Explanation (frequently used vs infrequently used)	0.94 (0.09)		2.56 (2.16-3.04)		<.001	
	Assurance (used vs not used)	1.28 (0.17)		3.61 (2.57-5.06)		<.001	
	Recommendation (used vs not used)	0.27 (0.08)		1.31 (1.12-1.53)		<.001	
	Facilities (used vs not used)	0.57 (0.25)		1.78 (1.08-2.91)		.02	
	Patient-centered care (used vs not used)	−0.48 (0.09)		0.62 (0.52-0.74)		<.001	
	Professional advice (frequently used vs infrequently used)	0.60 (0.08)		1.81 (1.55-2.13)		<.001	
	Performance assessment (use vs no use)	0.77 (0.08)		2.17 (1.84-2.55)		<.001	
**Random effects**							
	Dentist-level variance	1.13 (0.13)		N/A		N/A	
	Variance partition coefficient	0.28		N/A		N/A	

^a^Odds ratio for the patient experiencing a positive encounter when words from the themes are present in the review.

^b^N/A: not applicable.

## Discussion

Eight themes were identified related to patient reviews of encounters with dental health professionals: 6 (75%) were linked to higher patient satisfaction scores and the experience of a positive dental encounter, while 2 (25%) were linked to lower patient satisfaction scores and the experience of a challenging dental encounter.

### Principal Findings

If words related to the themes *explanation*, *assurance*, *recommendation*, *performance assessment*, *facilities*, and *professional advice* were present, patients were more likely to rate their experience as satisfying. Conversely, the use of words from the themes *consequences of treatment need* and *patient-centered care* reduced the likelihood of patients rating their experience as satisfying. In the following subsections, we will discuss the results and their implications.

Challenging encounters have been defined earlier in this paper as situations resulting in aversive feelings for dental health professionals [7] and as encounters involving conflicts between the perspectives of dental health professionals and those of the dental patient. In this study, we were interested in the challenging encounter from the dental patients’ perspectives, and it was assumed that when patients provided low satisfaction ratings, it indicated the experience of a challenging encounter. Conversely, high satisfaction ratings, it was assumed, indicated the absence of a challenging encounter.

### Consequences of Treatment Need

The theme *consequences of treatment need* contained words that could be interpreted as a reflection of unmet expectations, and we found that this theme was associated with an increased probability of experiencing a challenging encounter. Previous research suggests that some patients might have unrealistic expectations of dental treatment outcomes [38], and it is likely that a disjunction between treatment expectations and perceived treatment outcomes could influence the satisfaction with dental treatment, as indicated by a previous study [19]. However, Yao et al [38] suggest that the studies investigating dental treatment expectations in relation to patient satisfaction do not properly define the term “expectations” and that the results from the studies are diverse and difficult to interpret. This is supported by a recent literature review, which found it difficult to propose a link between patient satisfaction and expectations and suggested that this could be because “expectations” as a concept is not consistently defined in the literature and furthermore that it might be only indirectly associated with patient satisfaction [9].

On the basis of the words used in the *consequences of treatment need* theme, we found that the patients discussed reasons to seek dental health care (eg, “pain” and “caries”), as well as challenges that might have developed (eg, complications and disappointment regarding the outcome). In addition, the patients sometimes expressed feelings that could indicate a lack of self-agency (eg, words and expressions such as “had to,” “because,” and “caries”), which would indicate that the patients felt that they *had* to see the dentist because of a dental issue or some external cause. Motivations for seeking dental treatment could play a major role in how patients experience the dental treatment. One could envision that the dental encounter would be experienced differently based on the source of the patient’s motivation (eg, based on the motivational locus: internal vs external). This closely resembles key features of a problem-oriented visiting pattern, which refers to patients only seeking dental help when faced with acute circumstances (eg, pain or fractured teeth). This type of attendance has been associated with increased risk of tooth loss [39] and reduced oral health–related quality of life [40] compared to regular attendance. A Finnish study investigating dental patients’ perception of their dentist’s explanation during treatment found that patients with a problem-oriented visiting pattern and that perceived their economic situation as difficult were more likely to feel dissatisfied with their dentist’s explanation skills [41]. It has also been found that individuals with a fear of dental treatment tend to delay treatment and more often report poor oral health [42,43], implying that these patients could often have a problem-oriented visiting pattern. Therefore, it is not very surprising that this theme increases the risk of experiencing a challenging encounter. This could have been avoided if the patients had visited their dentist more often. Different intervention strategies have been used aimed at motivating patients to visit their dentist regularly (eg, community-based dental campaigns and a reduction in expenses) [44]. A recent literature review found that regular attendance could be increased if patients had the opportunity to visit a dental anxiety clinic and receive dental check-ups for free [44]. Such interventions could prove valuable to reduce challenging encounters in the clinic from the patient’s perspective.

### Patient-Centered Care

The theme *patient-centered care* was related to lower satisfaction with the dental encounter. Initially, this might seem odd because we would expect patient centeredness in dental health care to be a positive element. However, in this case, we would argue that the patients would primarily use words related to this theme when they discuss the lack of patient centeredness, which could again be similar to the notion of unmet expectations. In any case, it is clear that the patients in this study are concerned with patient centeredness in a dental context, which is in support of other findings suggesting that dentists need to improve their communication skills and be empathetic when cooperating with patients [45]. Furthermore, research has shown that dental students' self-reported empathy may diminish with increased patient interaction [46]. Even so, a study investigating a patient-centered training program and its effects on dental students’ self-reported empathy has revealed promising results to halt this concerning trend [47]. Other research implementing communication training programs in dentistry show that applying active training methods, such as role play and patient treatment experience, as well as acquiring behavioral or psychological knowledge alongside attending more traditional didactic lectures, was most effective in improving dental students’ communication skills [48].

### Assurance and Explanation

The theme *assurance* was associated with higher patient satisfaction, supporting results from other studies that have proposed a link between higher patient satisfaction and the perception of caring or comforting staff behavior [21]. This supports the idea that in dental encounters, patients might be in need of assurance and comforting behaviors because they might perceive that they have little control over the situation. The establishment of trust between the dentist and the patient has long been regarded as an essential part of treatment, with a corresponding impact on treatment outcomes [49]. Therefore, behaviors associated with *assurance* could help prevent a challenging encounter. It has been found too that when patients perceive their dentists’ *explanation* skills as good, they indicate greater satisfaction with treatment [50,51]. The relationship among the dental health professionals involved in the treatment seems to influence patient satisfaction [52], as well as the dental assistant’s knowledge of the patient’s needs [51]. Investing time in careful explanations before and during treatment could be a useful way to prevent challenging encounters and increase patient satisfaction.

### Professional Advice

Dentists have a professional responsibility to teach patients how to take care of their oral health. The theme *professional advice* could be interpreted as the patients’ perception of this teaching practice. It could also be viewed as proof that patients welcome professional advice regarding how to take care of their oral health. Oral health literacy is the individual’s ability to obtain, understand, and use oral health information [53,54]. According to a recent literature review, it consists of three important aspects: (1) the individual’s capacity to access health information through basic information acquisition skills (eg, the ability to read, an understanding of numbers, and the capability to interpret facial expressions), (2) the individual’s ability to use the information (eg, informed decision-making), and (3) oral health maintenance abilities (eg, self-regulation and goal achievement) [55]. The patient’s perception of the dentist’s ability to communicate and provide useful information about the patient’s oral health is therefore dependent not only upon the skills of the dentist but also on the patient’s oral health literacy. Dentists should consider that health information can be difficult to access and that information should be individually adapted according to patients’ abilities.

### Recommendation, Performance Assessment, and Facilities

Not surprisingly, the patients who wrote about positive dental encounters used more words related to *recommendation*, which suggests a need to disseminate their view of the dentist to peers on the web. Other research has supported the existence of this need, where web-based review sites are used to disseminate experiences and views to peers [20]. This sharing of experiences is believed to have a more profound meaning to users than can sometimes be suspected because shared experiences can function as a gateway to feeling connected to others and feeling empowered as a user of health care services [56]. In addition, *performance assessment* was associated with a higher satisfaction rating. This is supported by previous research findings linking patients’ perception of high-quality performance to increased patient satisfaction [21,57]. Dental health professionals could benefit from continuous training in clinical skills and striving to update their knowledge according to medical advances. The theme *facilities* was linked to a small increase in odds that the patient was satisfied, which extends the prior finding that patients seemed to write about clinical facilities in both positive encounters and challenging encounters [26] and that this theme was seemingly independent of the satisfaction rating. However, high-quality facilities have been linked to higher patient satisfaction in previous studies [21,58]. As some of these studies were conducted with inpatients at hospitals, it could be the case that patients needing to stay longer at the clinic found high-quality facilities to be more important for overall satisfaction.

### Strengths and Limitations

A LIWC analysis is best performed when the word count in each sample text exceeds 50 [30]. As previously stated, LIWC dictionaries work by calculating the relative percentage of a word’s occurrence in a body of text. In samples with small text sizes, for example 5 to 10 words, the relative percentage of each word tends to be very high; for example, in the sentence “I was at Molly’s birthday,” we see that the word “birthday” accounts for 20% of the word use. To counteract this effect, Boyd [30] suggests that one could have a sample size that is very large. For dental patients’ reviews to be accepted on the previously mentioned Norwegian website, they need to be at least 100 characters long [59]. We would argue that, in this case, since the mean word count is close to 50 (mean 48.9, SD 39) and the sample size is large (n=11,764), our findings will be less affected by this bias. However, it could prove valuable to repeat this study using larger sample sizes. As a language analysis tool, the LIWC has proven to be reliable in research [29], with examples available from a wide range of research to underscore its usefulness [60,61].

In general, it would be expected that only a limited number of patients would write a web-based review after a visit to the dentist. A true estimate of the response rate is not possible because we do not know the exact number of patients who have chosen not to respond or whether a patient has provided ratings for several dentists. Given the low review volume relative to the dentist-to-patient ratio (1:1250 [62]) and a mean of 3.9 reviews per dentist, only a small percentage of patients likely write online reviews. However, this should not significantly impact our ability to investigate themes related to high versus low patient satisfaction, which was our study objective. Because most of the reviews were positive (10,977/11,764, 93.31% have a rating of 4-5 stars), this could mean that the findings in our study are more representative of positive reviews. To counteract this bias, one could consider splitting the data set into 2 parts before analysis: the reviews with a low satisfaction rating (1-2 stars) versus the reviews with a high satisfaction rating (4-5 stars). This approach would enable a separate word analysis for each data set to compare the satisfied patients versus the unsatisfied patients and their word use. However, the number of reviews representing a low satisfaction rating was considered to be insufficient to provide reliable results in a bottom-up text analysis using the MEM, which usually depends on a large amount of text data to provide reliable results.

In the exploratory factor analysis performed using SPSS software, the words within each word category were extracted from the body of text based on how often they appeared together in a phrase. The interpretation and labeling of the themes were based on a theoretical understanding of the meaning of the factors. Other researchers intending to perform similar analyses could arrive at different theme labels based on their particular theoretical understanding; for instance, the theme *consequences of treatment need* was a broad category containing a greater number of words than the other themes, and we found it difficult to interpret and to agree on the final label because it seemed to be a theme with multiple layers. By contrast, other themes containing fewer words were more easily interpretable (eg, the themes *facilities* or *assurance*). This is a limitation related to the use of factor analysis often mentioned in the literature [63]. Despite these challenges, previous research has arrived at themes that are similar in their content with regard to patient satisfaction [21,57], indicating that our findings could be applicable in other contexts.

### Implications for Future Research

Websites provide large amounts of text data that will enable researchers to perform large-scale analyses (eg, using text analysis programs that build upon machine learning methods, such as BERT [64]). Even so, machine learning methods could encounter difficulties related to “poor language” in short internet texts, elucidating the need to develop these methods further [65]. The findings from this study and similar studies could help clinicians develop a better understanding of their patients’ perspectives and needs in light of challenging treatment situations. Hopefully, some of these findings could also help guide future research on increasing patient satisfaction, while limiting challenging encounters in the dental clinic. In addition, there is a need to establish effective interventions to motivate patients to visit their dentist regularly.

### Conclusions

The findings of this study demonstrate the value of web-based patient reviews as a gateway to patient experiences, and we would argue that implementing the themes or elements from the themes expressed in these reviews could help improve patient satisfaction. While dissatisfaction with dental treatment seems to be associated with negative consequences and (a lack of) patient centeredness, high satisfaction seems to hinge on patients’ experiences of being acknowledged by the dentist. Investigations of web-based reviews could produce valuable insights into what patients experience and value in dental treatment settings.

## Appendix

Multimedia Appendix 1 Lemmatization list.

Multimedia Appendix 2 Stop list.

## Abbreviations

LIWC: Linguistic Inquiry and Word Count
MEM: meaning extraction method
NLP: natural language processing
OR: odds ratio
VPC: variance partition coefficient
